# Undiagnosed Kidney Injury in Uninsured and Underinsured Diabetic African American Men and Putative Role of Meprin Metalloproteases in Diabetic Nephropathy

**DOI:** 10.1155/2018/6753489

**Published:** 2018-04-29

**Authors:** Lei Cao, Rashin Sedighi, Ava Boston, Lakmini Premadasa, Jamilla Pinder, George E. Crawford, Olugbemiga E. Jegede, Scott H. Harrison, Robert H. Newman, Elimelda Moige Ongeri

**Affiliations:** ^1^Department of Biology, North Carolina A&T State University, Greensboro, NC 27411, USA; ^2^Cone Health Community Health and Wellness Center, Greensboro, NC 27401, USA

## Abstract

Diabetes is the leading cause of chronic kidney disease. African Americans are disproportionately burdened by diabetic kidney disease (DKD) and end stage renal disease (ESRD). Disparities in DKD have genetic and socioeconomic components, yet its prevalence in African Americans is not adequately studied. The current study used multiple biomarkers of DKD to evaluate undiagnosed DKD in uninsured and underinsured African American men in Greensboro, North Carolina. Participants consisted of three groups: nondiabetic controls, diabetic patients without known kidney disease, and diabetic patients with diagnosed DKD. Our data reveal undiagnosed kidney injury in a significant proportion of the diabetic patients, based on levels of both plasma and urinary biomarkers of kidney injury, namely, urinary albumin to creatinine ratio, kidney injury molecule-1, cystatin C, and neutrophil gelatinase-associated lipocalin. We also found that the urinary levels of meprin A, meprin B, and two kidney meprin targets (nidogen-1 and monocytes chemoattractant protein-1) increased with severity of kidney injury, suggesting a potential role for meprin metalloproteases in the pathophysiology of DKD in this subpopulation. The study also demonstrates a need for more aggressive tests to assess kidney injury in uninsured diabetic patients to facilitate early diagnosis and targeted interventions that could slow progression to ESRD.

## 1. Introduction

Diabetic kidney disease (DKD) is the leading cause of end stage renal disease (ESRD). Minority ethnic groups in the United States (e.g., African Americans, Native Americans, and Hispanics) are disproportionately affected by DKD. A 2008 US Renal Data Systems survey showed that ESRD rates associated with diabetes are three times higher in African Americans than in their Caucasian counterparts, with the percentage of new cases of kidney disease attributed to diabetes among young African Americans currently standing at 43%. The prevalence of type 2 diabetes is related to nutrition and sedentary lifestyles [1], which promote obesity and are associated with the metabolic syndrome. The most recent data on obesity in the US indicate that 40% of the adult population is obese [2]. Even more alarmingly, the same study shows a rising prevalence of obesity among the young, with 18.5% of children in the United States being obese. This trend in obesity is likely going to further exacerbate the current diabetes epidemic. Furthermore, the prevalence of obesity is significantly higher among Hispanics and African Americans of all age categories [2]. Other previous studies had shown that African Americans have greater adjusted odds of having diabetes compared to Caucasian Americans [3]. Other factors contributing to health disparities in diabetes include disparities in healthcare resource allocation [1, 4], healthcare utilization [4], quality of diabetes care, perceived self-efficacy, and susceptibility genes [5]. Minority men are at a markedly elevated risk for the receipt of low-quality healthcare. Studies have shown that the differences in ESRD care that African American and Caucasian American men have received are statistically significant [6], with African American men consistently receiving worse care.

While susceptibility genes are known to contribute to the disparities in DKD, the cellular and molecular mechanisms involved in its progression are not fully understood. Such knowledge is important for the development of therapies and diagnostic tools that are efficacious for people from diverse ethnic backgrounds. Recently, several protein biomarkers have been developed that may offer insights into the molecular mechanisms of disease pathology. These include the type I transmembrane glycoprotein, kidney injury marker-1 (KIM-1), the cysteine-protease inhibitor, cystatin C, and the ubiquitous lipocalin family member, neutrophil gelatinase-associated lipocalin (NGAL) [7–9]. Meprin zinc metalloproteases, which are abundantly expressed in the brush border membranes (BBM) of renal proximal tubules, have also emerged as susceptibility markers for DKD [10]. Meprins are also expressed in podocytes [11] and leukocytes (monocytes and macrophages) [12] and play a role in inflammation, an underlying cause of fibrosis as observed in DKD. Meprins are composed of two subunits, *α* and *β*, encoded by distinct genes on chromosomes 6 and 18, respectively, in humans [13, 14], and on chromosomes 17 and 18, respectively, in mice [15]. Oligomerization of meprins results in two protein isoforms, meprin A (*α*-*α* or *α*-*β*) and meprin B (*β*-*β*). Meprin *β* gene polymorphisms were associated with DKD in the Pima Indians, an ethnic group in the United States with an extremely high incidence of type 2 diabetes and subsequent ESRD [10]. Interestingly, both the expression and the activity of meprins decrease at the onset of diabetic kidney injury in rodent models [16].

Consistent with how decreased meprin activity relates to kidney injury, we recently showed that meprin* αβ* double knockout mice exhibit more severe kidney injury upon streptozotocin-induced type 1 diabetes [17].* In vitro* and* in vivo* studies have identified several kidney meprin targets that play a role in renal fibrosis. For example, meprins are capable of cleaving and/or degrading several extracellular matrix (ECM) proteins, such as procollagen III, collagen IV, laminin, fibronectin, and nidogen-1 [18–22]. Since renal pathological changes seen in DKD are a direct consequence of accumulation of ECM proteins [23–26] due to excess production and/or reduced degradation of ECM proteins, meprins could play a role in modulating this imbalance. Other studies have shown that meprins proteolytically process proteins involved in inflammation, an underlying process for renal fibrosis [27]. Such modulators of inflammation include proinflammatory cytokines (e.g., interleukin 1*β* (IL-1*β*) [28], IL-6 [29, 30], IL-18 [31]) and monocyte chemoattractant protein-1 (MCP-1 [32]) as well as the proteolytic release of the anti-inflammatory molecule, N-acetyl-seryl-aspartyl-lysyl-proline (Ac-SDKP) from thymosin *β*4 [33]. The objectives of the current study were to use recently developed protein biomarkers to evaluate undiagnosed kidney injury among uninsured and underinsured African American men in Greensboro, NC. We further sought to determine whether the levels of urinary meprins and their targets correlate with the existence and/or the severity of kidney injury.

## 2. Materials and Methods

### 2.1. Subjects

Diabetic African American men aged 18–65 years were recruited through the Cone Health Community Health and Wellness Center in Greensboro, North Carolina, a facility that primarily serves uninsured and underinsured patients. This study was approved by the North Carolina A&T State University and Cone Health Institutional Review Boards (IRB). Written informed consent was obtained from each study participant. Age-matched, nondiabetic controls were also recruited through the Community Health and Wellness Center and local faith-based organizations. Three groups were included: (i) diabetic patients without known kidney disease (*n* = 76); (ii) diabetic patients with diagnosed kidney disease (*n* = 21); and (iii) age-matched nondiabetic controls (*n* = 75). Surveys were administered to establish patient profiles and family medical history. Medical information was provided by participants through surveys and verified using medical records. Patients with ESRD and other chronic disease conditions were excluded. All patient data were deidentified prior to analysis.

### 2.2. Determination of Anthropometric Data Related to the Metabolic Syndrome

The height and body weight of each participant were measured during their visit and used to compute the body mass index. Additionally, blood pressure and waist circumference were measured.

### 2.3. Collection of Blood and Urine Samples

Fasting blood and urine samples were collected from the three groups of patients. The diabetic status for the nondiabetic control group was confirmed by measuring fasting glucose levels using a glucose meter (ReliOn®). Blood samples were obtained by trained phlebotomists via intravenous route, collected into heparin tubes, and stored on ice for an average of one hour before being processed to obtain plasma. To obtain plasma, the blood samples were centrifuged at 2,750 ×g for 15 minutes at 4°C using an Allegra X-14R centrifuge (Beckman Coulter, Brea, CA). The plasma was then aliquoted into microfuge tubes and stored at −80°C until proteomic analysis. Urine samples were also held on ice before being aliquoted and stored at −80°C until analysis.

### 2.4. Assessment of Kidney Injury

For biochemical assessment of kidney injury, we performed assays for traditional biomarkers of kidney injury, namely, urinary albumin and creatinine, which were then used to calculate the urinary albumin to creatinine ratio (UACR). The UACR is the current gold standard for clinical diagnosis of DKD. Albumin assays utilized Albuwell® ELISA kits from Exocell (Philadelphia, PA) while creatinine was measured using a calorimetric assay kit from Diazyme Laboratories (Poway, CA). We also determined the levels of three recently developed protein markers of kidney injury, namely, KIM-1, cystatin C, and NGAL using enzyme-linked immunosorbent assay (ELISA) (R&D Systems, Minneapolis, MN). All of the assays were performed according to the manufacturers' instructions with absorbance being read at 450 nm using a F500 Pro multimode microplate reader (Tecan, USA). Standard curves for all biomarkers except albumin were generated using four parameter logistic (4-PL) curve fits (GraphPad Prism software). Standard curves for albumin were generated using log-log regression, according to the manufacturer's instructions. The urinary levels of each kidney injury marker were normalized to the urinary creatinine levels.

### 2.5. Western Blot Analysis for Urinary Meprins and Nidogen-1

Western blot analysis was used to determine the urinary levels of meprin A, meprin B, and nidogen-1, according to previously described protocols [34–36]. Briefly, 25 *μ*l of the urine samples were combined with SDS loading buffer supplemented with *β*-mercaptoethanol and heated at 80°C for 5 minutes. Three or four representative samples from each group were then resolved on the same 8% polyacrylamide gel under denaturing conditions. Following electrophoresis, the proteins were transferred to a nitrocellulose membrane, blocked in blocking buffer (5% nonfat dry milk dissolved in tris-buffered saline, 0.05% Tween-20 (TBS-T)), and then probed with primary antibodies for meprin A (HMC14, rabbit polyclonal, diluted 1 : 3300 in blocking buffer), meprin B (HMC77, rabbit polyclonal diluted 1 : 5000 in blocking buffer), or nidogen-1 (Millipore; rat polyclonal diluted 1 : 1000 in blocking buffer). The HMC14 and HMC77 antibodies were a gift from Dr. Judith Bond (Penn State Hershey Medical Center). Following incubation with the primary antibody solution, membranes were washed three times in TBS-T for 15 min each and then incubated with corresponding secondary antibodies conjugated to horse radish peroxidase (Bio-Rad, diluted 1 : 10,000 in TBS-T). Finally, membranes were washed three times in TBS-T for 15 min each before the addition of chemiluminescence substrate (Thermo Scientific, Waltham, MA). Protein bands were detected by exposure to X-ray film or by exposure of the membrane to Amersham Imager 600 (GE Healthcare, Chicago, IL). The Western blots were repeated for a total of 9 or 12 samples from each subcategory. We first grouped samples based on self-reported and diagnosed diabetes status, with 4 representative samples from each group (diabetics, diabetics with DKD, and nondiabetic controls) included in each gel. Subsequent blots grouped the samples from diabetic patients without diagnosed kidney disease based on their UACR levels, with 3 samples from each group included in each gel, that is, (i) nondiabetic, (ii) normoalbuminuria (UACR < 30 mg/g), (iii) microalbuminuria (30 mg/g ≤ UACR ≤ 300 mg/g), and (iv) macroalbuminuria (UACR > 300 mg/g).

### 2.6. Assays for MCP-1

We performed assays for a second meprin target, monocyte chemoattractant protein 1 (MCP-1), using ELISA kits (R&D Systems) according to the manufacturer's instructions. Standard curves generated using 4-PL curve fits (GraphPad Prism) were used to determine the MCP-1 concentrations. Urinary MCP-1 levels were normalized to the urine creatinine levels in each sample.

### 2.7. Statistical Analysis

The data were analyzed by one-way ANOVA and Tukey's honest significant difference test (GraphPad Prism Software). Logarithmic transformation was conducted for the kidney injury biomarker values (i.e., UACR, KIM-1, Cystatin C, NGAL) and MCP-1 before statistical analysis. To identify the diabetic subjects with high risk for kidney injury, the mean, standard deviation, upper quartile (Q3), and interquartile range (IQR) from the nondiabetic group were calculated. Diabetic subjects with values greater than mean + 2SD and Q3 + 1.5IQR of the nondiabetic group were considered to be at high risk for DKD.

## 3. Results

### 3.1. Anthropometric Data Related to the Metabolic Syndrome

**Figure 1:**
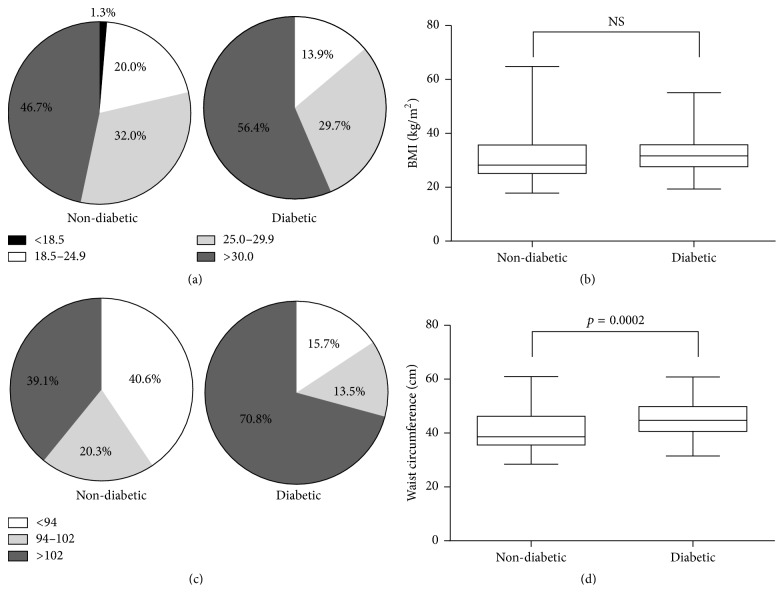
*Anthropometric data related to the metabolic syndrome.* (a) BMI distribution in nondiabetic and diabetic groups showing the proportion of underweight (BMI < 18.5 kg/m^2^), normal weight (18.5 kg/m^2^ ≤ BMI ≤ 24.9 kg/m^2^), overweight (25.0 kg/m^2^ ≤ BMI ≤ 29.9 kg/m^2^), and obese (30.0 kg/m^2^ ≤ BMI) in nondiabetic (*n* = 75) and diabetic (*n* = 85) groups. (b) Box plots of BMI for nondiabetic and diabetic groups. The upper and lower whiskers indicate the maximum and minimum values. The upper and lower borders of the box indicate the 25th and the 75th percentile, respectively. The black line in each box indicates the median. (c) Waist circumference (WC) distribution in nondiabetic and diabetic groups showing the proportion of normal waist circumference (WC < 94 cm), increased health risk (94 cm ≤ WC ≤ 102 cm), and substantial health risk (102 cm < WC) in nondiabetic (*n* = 69) and diabetic (*n* = 85) groups. (d) Box plots of WC for nondiabetic and diabetic groups. The upper and lower whiskers indicate the maximum and minimum values, respectively. The upper and lower borders of the box indicate the 25th and the 75th percentile, respectively. The black line in each box indicates the median. NS indicates no significant difference.

**Figure 2:**
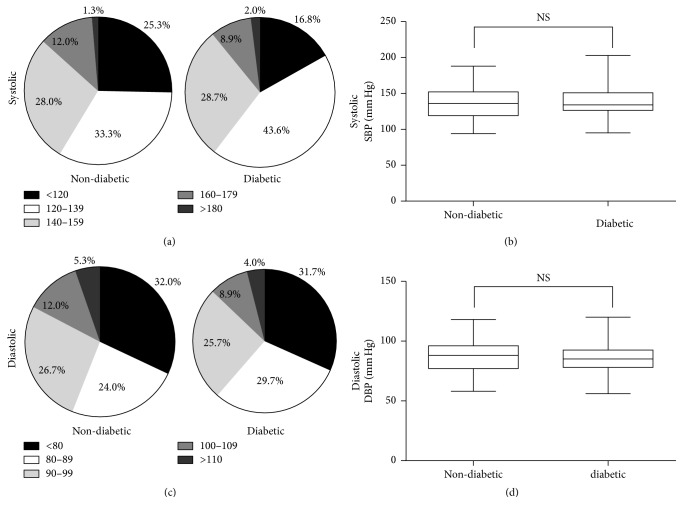
*Blood pressure measurements.* (a) Systolic blood pressure distribution in nondiabetic and diabetic groups showing the proportion of normal BP (80 mm Hg ≤ SBP < 119 mm Hg), prehypertension (120 mm Hg ≤ SBP ≤ 139 mm Hg), hypertension stage 1 (140 mm Hg ≤ SBP ≤ 159 mm Hg), and hypertension stage 2 (SBP ≤ 160 mm Hg) in nondiabetic (*n* = 69) and diabetic (*n* = 85) groups. (b) Box plot of systolic blood pressure for nondiabetic and diabetic groups. The upper and lower whiskers indicate the maximum and minimum values, respectively. The upper and lower borders of the box indicate the 25th and the 75th percentile, respectively, while the black line in each box indicates the median. (c) Diastolic blood pressure distribution in nondiabetic and diabetic groups showing the proportion of normal BP (60 mm Hg < DBP < 79 mm Hg), hypertension (80 mm Hg < DBP < 89 mm Hg), hypertension stage 1 (90 mm Hg ≤ DBP ≤ 99 mm Hg), and hypertension stage 2 (DBP ≤ 100 mm Hg). (d) Box plot of diastolic blood pressure for nondiabetic and diabetic groups. The upper and lower whiskers indicate the maximum and minimum values, respectively. The upper and lower borders of the box indicate the 25th and the 75th percentile, respectively. The black line in each box indicates the median. SBP: systolic blood pressure. DBP: diastolic blood pressure. NS indicates no significant difference.

Since the metabolic syndrome is often associated with diabetes mellitus, we first assessed various measures of obesity and cardiovascular health among members of each group. Based on BMI measurements, the majority of the participants in our study were either overweight (25.0 ≤ BMI ≤ 29.9) or obese (BMI ≥ 30), with no significant differences between nondiabetic and diabetic participants (Table 1 and Figures 1(a) and 1(b)). On the other hand, significant differences were observed in the mean waist circumference of diabetic and nondiabetic patients (Table 1 and Figure 1(d); p=0.0002). Notably, a substantially larger proportion of diabetic patients (70.8%) had a waist circumference > 102 cm compared to nondiabetic patients (39.1%) (Figure 1(c)). Finally, 77.0% of nondiabetic and 85.1% of diabetic participants were prehypertensive (120 mm Hg ≤ systolic bp ≤ 139 mm Hg or 80 mm Hg ≤ diastolic BP ≤ 89 mm Hg), hypertensive stage 1 (140 mmHg ≤ systolic BP ≤ 159 mm Hg or 90 mm Hg ≤ diastolic BP ≤ 99 mm Hg), or hypertensive stage 2 (systolic BP ≥ 160 mm Hg, or diastolic BP ≥ 100 mm Hg) (Table 1 and Figures 2(a)–2(d)).

### 3.2. Undiagnosed Kidney Injury in Diabetic Patients

**Figure 3:**
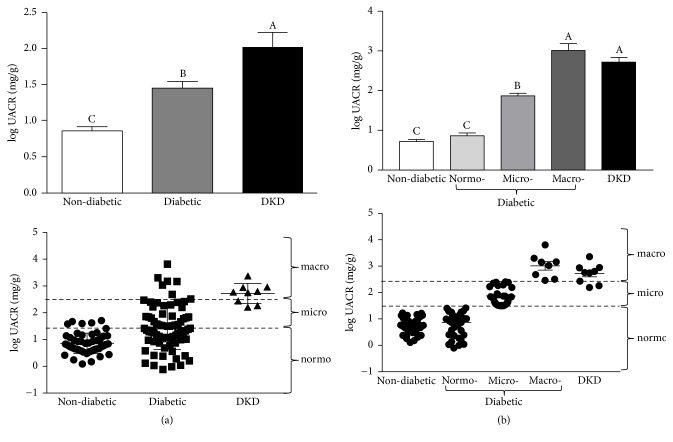
*Urinary albumin to creatinine ratios (UACRs).* (a) Log-transformed UACRs among nondiabetic controls, diabetic patients with no known kidney disease, and diabetic patients with diagnosed diabetic kidney disease (DKD). Data are represented as both a bar chart showing mean log UACR ± SEM within each group (top) and a scatter plot showing values for each individual within the group (bottom). UACR levels corresponding to normo- (log⁡UACR < 1.47), micro- (1.47 < log⁡UACR < 2.47), and macroalbuminuria (log⁡UACR > 2.47) are indicated by dashed lines. (b) Log-transformed UACR as in (a), except that diabetic patients with no known kidney disease have been subdivided into normo-, micro-, and macroalbuminuria based on their UACRs. Labels without a common letter are significantly different from one another (*p* < 0.05) based on one-way ANOVA and Tukey's honest significant difference test.

**Figure 4:**
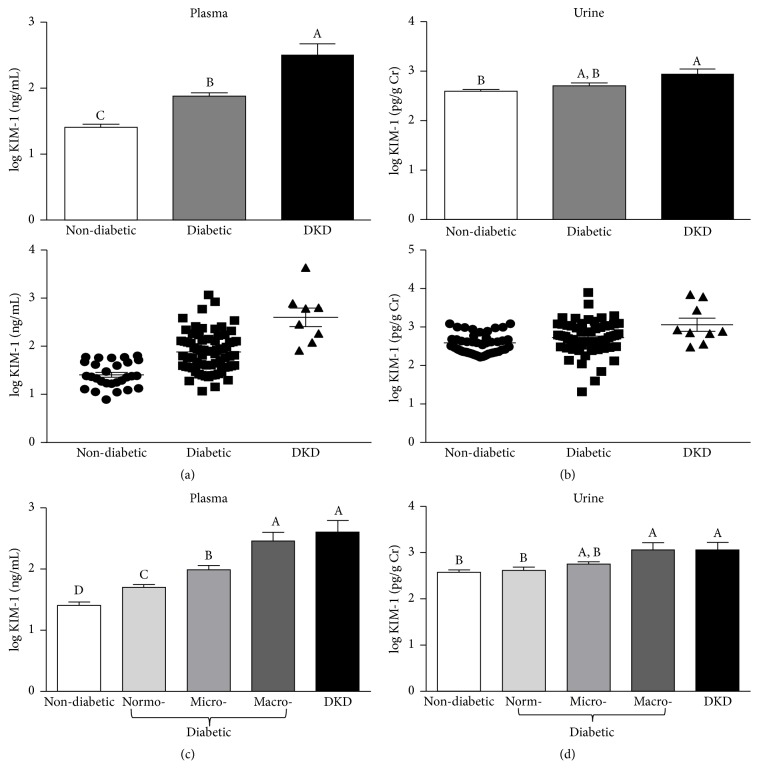
*Plasma and urinary kidney injury molecule-1 (KIM-1).* (a)-(b) Log-transformed plasma (a) and urinary (b) KIM-1 levels among nondiabetic subjects, diabetic patients with no known kidney disease (diabetic), and patients with diagnosed diabetic kidney disease (DKD). Urinary KIM-1 levels were normalized to creatinine to account for differences in urine concentration. Data are represented as both a bar chart showing mean log⁡KIM-1 ± SEM within each group (top) and a scatter plot showing values for each individual within the group (bottom). (c)-(d) Log-transformed plasma (c) and urinary (d) KIM-1 levels among nondiabetic subjects, diabetic patients with normo-, micro, or macroalbuminuria, and patients with diagnosed diabetic kidney disease (DKD). Urinary KIM-1 was normalized to urinary creatinine. Data are represented as mean ± SEM. Labeled means without a common letter are significantly different from one another (*p* < 0.05) based on one-way ANOVA and Tukey's honest significant difference test.

**Figure 5:**
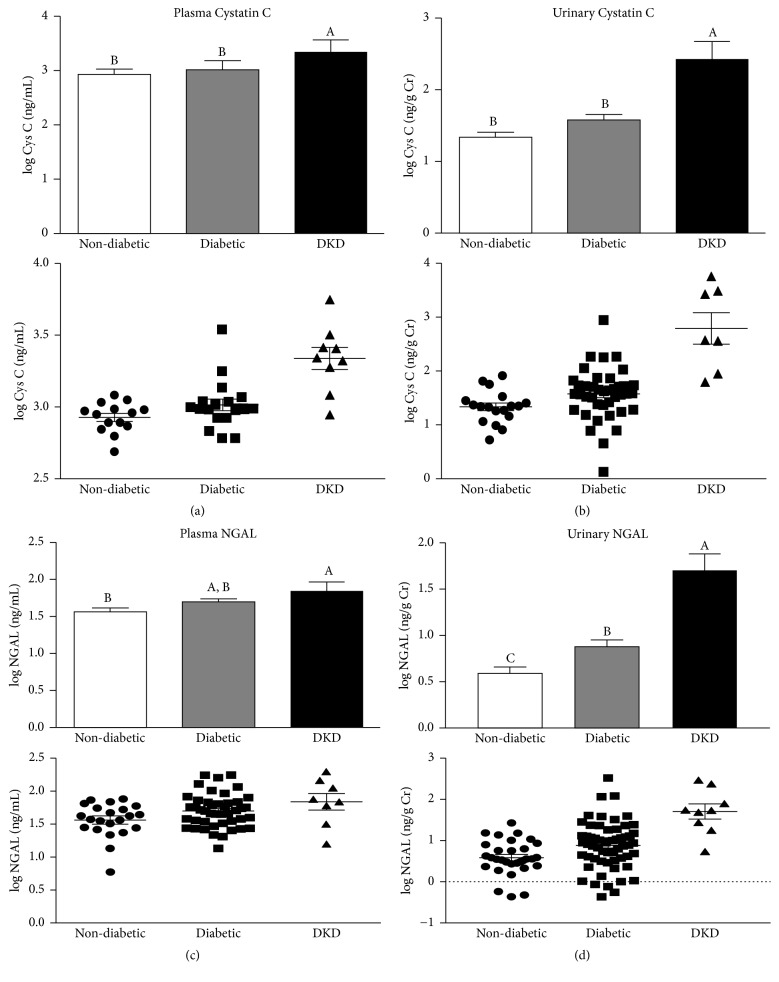
*Cystatin C and neutrophil gelatinase-associated lipocalin (NGAL) levels based on clinical diagnosis status.* (a)-(b) Log-transformed plasma (a) or urinary (b) cystatin C levels among nondiabetic subjects, diabetic patients with no known kidney disease (diabetic), and patients with diagnosed diabetic kidney disease (DKD). Urinary cystatin C levels were normalized to urinary creatinine to account for differences in urine concentration. Data are represented as both a bar chart showing mean log Cystatin C ± SEM within each group (top) and a scatter plot showing values for each individual within the group (bottom). (c)-(d) Log-transformed plasma (c) and (d) urinary NGAL levels among nondiabetic subjects, diabetic patients with no known kidney disease (diabetic), and patients with diagnosed diabetic kidney disease (DKD). Urinary NGAL levels were normalized to urinary creatinine to account for differences in urine concentration. Data are represented as both a bar chart showing mean log NGAL ± SEM within each group (top) and a scatter plot showing values for each individual within the group (bottom). Labeled means without a common letter are significantly different from one another (*p* < 0.05) based on one-way ANOVA and Tukey's honest significant difference test.

**Figure 6:**
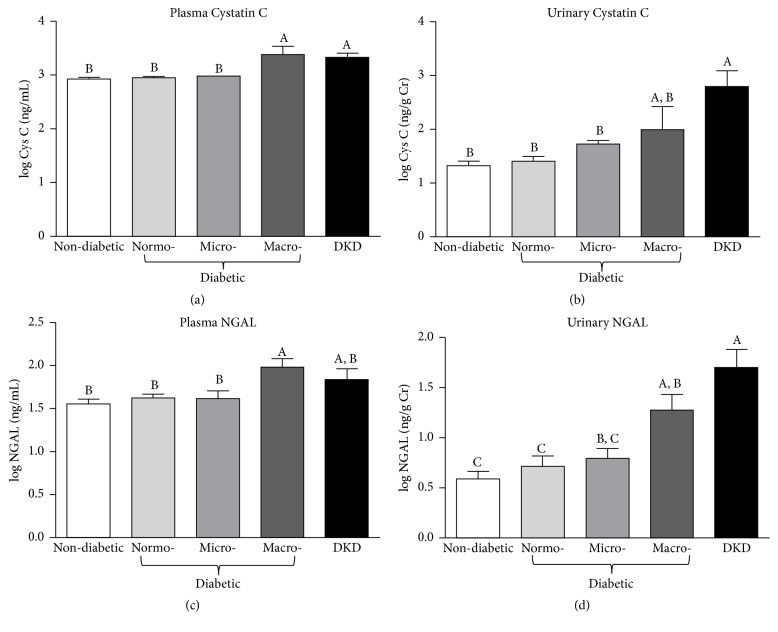
*Cystatin C and neutrophil gelatinase-associated lipocalin (NGAL) levels.* (a)-(b) Log-transformed plasma (a) or urinary (b) cystatin C levels among nondiabetic subjects, diabetic patients with normo-, micro- or macroalbuminuria, and diabetic patients with diagnosed kidney disease (DKD). Urinary cystatin C levels were normalized to urinary creatinine. (c)-(d) Log-transformed plasma (c) and urinary (d) NGAL levels among nondiabetic subjects, diabetic patients with normo-, micro- or macroalbuminuria, and diabetic patients with diagnosed kidney disease (DKD). Urinary NGAL levels were normalized to urinary creatinine. Data are represented as mean ± SEM. Labeled means without a common letter are significantly different from one another (*p* < 0.05) based on one-way ANOVA and Tukey's honest significant difference test.

To assess the incidence of kidney injury within each group, we first determined the UACR for each participant based on his albumin and creatinine levels (Figure 3(a)). While the average UACRs of both the diabetic patients and the patients with diagnosed DKD were significantly higher than those of the nondiabetic controls, the average UACR in the diabetic group was significantly lower than that of patients with diagnosed DKD (*p* = 0.01 after transformation). Interestingly, there was a large degree of variation in UACRs among the diabetic patients, suggesting that members of this group may exhibit varying degrees of kidney injury (Figure 3(a), bottom panel). Therefore, in subsequent analyses, we used the UACRs to further subdivide the diabetic participants without known kidney disease into three subgroups: normoalbuminuria (UACR < 30 mg/g); microalbuminuria (30 mg/g ≤ UACR ≤ 300 mg/g); and macroalbuminuria (UACR > 300 mg/g) (Figure 3(b)). Among the diabetic patients, 54.0% (41/76) had an UACR characteristic of normoalbuminuria, 35.5% (27/76) had UACR indicative of microalbuminuria, and 10.5% (8/76) had UACR in the macroalbuminuria range. Moreover, five of the participants in the microalbuminuria subgroup (5/27, 18.5%) exhibited UACR levels between 200 and 300 mg/g, placing them near the borderline between micro- and macroalbuminuria. This distribution suggested that a significant proportion of the diabetic patients may have varying degrees of undiagnosed kidney injury. Therefore, we used a series of recently developed proteomic markers of kidney injury to further interrogate the kidney injury status of the participants (Figures 4–6). For instance, KIM-1 is a type I transmembrane glycoprotein that has recently been correlated with kidney tissue damage in models of acute kidney injury as well as DKD [37–39]. Analysis of plasma KIM-1 levels revealed that diabetic patients had significantly higher plasma KIM-1 levels (*p* < 0.0001) than nondiabetic controls (Figure 4(a)). Moreover, when plasma KIM-1 values were compared between the diabetic and nondiabetic groups, 52% of the diabetic subjects had plasma KIM-1 values that were at least two standard deviations above the mean of the nondiabetic group (Table 2). Perhaps more strikingly, 45% of the patients in the diabetic group exhibited plasma KIM-1 levels that were three standard deviations above the mean, further suggesting that a large proportion of the patients in the diabetes group may be suffering from undiagnosed kidney disease. Indeed, comparison among diabetic patients with varying UACR levels revealed significant differences between the subgroups (Figure 4(c)). In fact, plasma KIM-1 levels appear to correlate with severity of kidney injury (as determined by UACR), with diabetic patients with macroalbuminuria exhibiting significantly higher levels than diabetic patients with microalbuminuria, which exhibited significantly higher levels than diabetic patients with normoalbuminuria, which exhibited significantly higher levels than nondiabetic controls (Figure 4(c)).

We next investigated whether urinary KIM-1 levels also correlated with the extent of kidney injury among the participants in our study. To account for differences in urine concentration, during these analyses, we normalized urinary levels of KIM-1 to the creatinine levels for each subject. Though normalized urinary KIM-1 levels were elevated in diabetic patients compared to the nondiabetic controls, there was not a significant difference between the two groups (Figure 4(b)). However, subcategorization based on UACR revealed that diabetic patients with macroalbuminuria exhibited normalized urinary KIM-1 levels similar to those within the DKD group (Figure 4(d)). Importantly, the levels observed in the macroalbuminuria group were significantly higher than those of either the nondiabetic controls or the diabetic patients with normoalbuminuria (Figure 4(d)). Moreover, though the proportion of high urinary KIM-1 values among diabetic subjects was less than that observed for plasma KIM-1, 23% and 12% of the diabetic patients still exhibited urinary KIM-1 levels that were two and three standard deviations above the mean of the nondiabetic controls, respectively (Table 2). A similar distribution was also observed if the comparison was done using the upper quartile plus either 1.5 times or 3 times the interquartile range (Table 2). Thus, KIM-1 appears to correlate well with the extent of kidney injury among the African American men in our study, with plasma KIM-1 showing a stronger correlation than urinary KIM-1.

Similar trends were also observed for two other emerging protein markers of kidney injury, namely, cystatin C and NGAL [38, 40]. For instance, both urinary and plasma cystatin C were significantly higher among patients with DKD compared to nondiabetic controls and diabetic patients without diagnosed DKD (Figures 5(a) and 5(b)). In contrast, only marginal increases were observed between the diabetic group and the nondiabetic controls (Figures 5(a) and 5(b)). However, within the diabetic group, patients with macroalbuminuria exhibited significantly higher plasma cystatin C levels than either nondiabetic controls or diabetic patients with normoalbuminuria and microalbuminuria (Figure 6(a)). On the other hand, though normalized urinary cystatin C levels increased steadily as patients in the diabetic group progressed from normoalbuminuria to microalbuminuria to macroalbuminuria, the observed increases did not result in urinary cystatin C levels that were significantly higher than those observed in the nondiabetic group (Figure 6(b)). Indeed, only patients in the DKD group exhibited significantly higher levels of normalized urinary cystatin C. Interestingly, the percentage of participants whose cystatin C levels were two standard deviations above the mean of the nondiabetic group was similar regardless of whether plasma or urinary cystatin C was considered (17% versus 19%, resp.) (Table 2). In contrast, when the interquartile range (IQR) was used to compare the groups, differences emerged between plasma and urinary cystatin C. For instance, while a similar percentage of subjects (17%) were found to be 1.5 IQR above the third quartile (Q3) when plasma cystatin C levels were used, a much larger percentage of subjects (43%) exhibited urinary cystatin C levels that were 1.5 IQR above Q3 (Table 2). Since IQR is more robust against outliers and nonnormal data, this may suggest that one or two outliers in the diabetic group may have resulted in a skewed or nonnormal distribution for the urinary cystatin C values. Like cystatin C, analysis of NGAL levels also revealed elevated levels in a subpopulation of the diabetic group. For example, though significantly higher levels of urine and plasma NGAL were only observed in patients with DKD when patients were grouped according to diabetic status alone (Figures 5(c) and 5(d)), closer examination revealed that diabetic patients with macroalbuminuria exhibited significantly higher NGAL levels than nondiabetic controls in both their plasma and urine (Figures 6(c) and 6(d)). The percentages of diabetic subjects having plasma and urinary NGAL levels greater than two SD above the mean of nondiabetic subjects were 17% and 24%, respectively (Table 2). A similar distribution (13% and 24% for plasma and urinary NGAL, resp.) was observed if IQR was used for comparison. Together, the assays for proteomic markers of kidney function revealed undiagnosed kidney injury in a significant proportion of the diabetic patients (Table 2). This was true for assays utilizing traditional measures of kidney injury, that is, UACR (Figure 3), as well as a panel of recently developed proteomic markers of kidney injury (Table 2; Figures 4–6).

### 3.3. Elevated Urinary Levels of Meprins and Meprin Targets in Patients with Diabetic Kidney Injury

**Figure 7:**
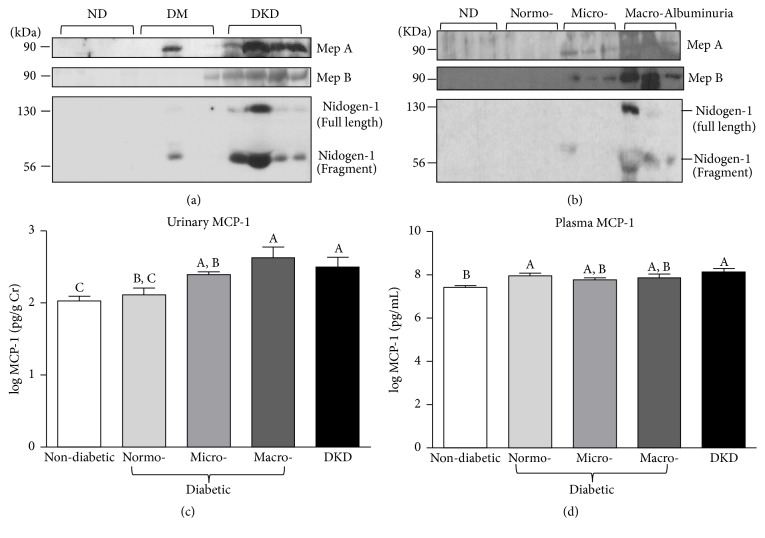
*Urinary meprin A, meprin B, nidogen-1, and monocyte chemoattractant protein-1 (MCP-1).* (a) Representative immunoblots for urinary meprin A, meprin B, and nidogen-1 grouped according to diabetes status; nondiabetic controls (ND), diabetic patients with no known kidney disease (DM), and patients with diagnosed diabetic kidney disease (DKD). (b) Representative immunoblots for meprin A, meprin B, and nidogen-1 in samples from nondiabetic controls (ND) and diabetic patients without known kidney disease grouped into normo-, micro-, and macroalbuminuria based on the UACR. (c)-(d) Log-transformed plasma (c) and urinary (d) MCP-1 levels among nondiabetic controls, diabetic patients with normo-, micro-, or macroalbuminuria, and patients with diagnosed diabetic kidney disease (DKD). Urinary MCP-1 was normalized to urinary creatinine. Data are represented as mean ± SEM. Labeled means without a common letter are significantly different from one another (*p* < 0.05) based on one-way ANOVA and Tukey's honest significant difference test.

**Figure 8:**
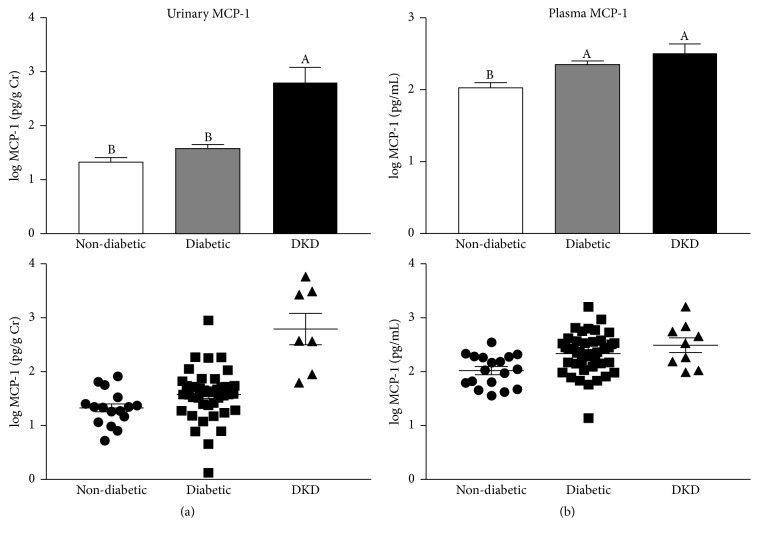
*Plasma and urinary monocyte chemoattractant protein-1 (MCP-1).* Log-transformed plasma (a) and urinary (b) MCP-1 levels among nondiabetic subjects, diabetic patients with no known kidney disease (diabetic), and patients with diagnosed diabetic kidney disease (DKD). Urinary MCP-1 levels were normalized to creatinine to account for differences in urine concentration. Data are represented as both a bar chart showing mean log⁡MCP-1 ± SEM within each group (top) and a scatter plot showing values for each individual within the group (bottom). Labeled means without a common letter are significantly different from one another (*p* < 0.05) based on one-way ANOVA and Tukey's honest significant difference test.

Next, to identify new biomarkers of DKD and to potentially gain mechanistic insights into disease progression, we asked whether the levels of meprins A and B correlated with DKD. When compared to nondiabetic controls, Western blot analysis revealed detectable levels of both meprin A and meprin B in diabetic patients with DKD and some diabetic patients without diagnosed kidney disease (Figure 7(a)). Importantly, similar to plasma KIM-1, the levels of urinary meprins were higher in patients with both micro- and macroalbuminuria, suggesting a positive correlation between urinary meprins and the severity of kidney injury (Figure 7(b)). The 90 kDa band corresponds to the expected size of shed monomeric meprins under reducing/denaturing conditions [41, 42]. To determine if the meprin levels were due to shedding or general damage to the proximal tubules, we probed for villin, a cytoskeletal protein that is highly expressed in the BBM of proximal tubules. There were no detectable levels of villin in the urine from any of the groups evaluated (data not shown). We also demonstrated that patients with DKD exhibited relatively high levels of two meprin targets, nidogen-1 and MCP-1. For instance, while nidogen-1 was undetectable in patients with UACR ≤ 300 mg/g, the levels were much higher in patients with UACR > 300 mg/g, increasing with severity of kidney injury (Figure 7(b)). Interestingly, a fragment of nidogen-1 migrating at ~50 kDa was also detectable in urine from diabetic patients with kidney injury as determined by UACR, perhaps corresponding to a cleavage product. A similar trend was also observed for urinary MCP-1, with levels being highest in patients with diagnosed DKD and diabetic patients with macroalbuminuria (Figures 7(c) and 8(a)). Interestingly, although both the normoalbuminuria and DKD groups are characterized by higher plasma MCP-1 levels than the nondiabetic group, there were no significant differences in plasma MCP-1 levels among the DKD and diabetic groups regardless of their UACR levels (Figures 7(d) and 8(b)). The observed increase in urinary MCP-1, but not plasma MCP-1, may suggest that the elevated urinary excretion of MCP-1 was caused by impaired kidney function.

## 4. Discussion

Diabetes is the leading cause of chronic kidney disease, a complication associated with high morbidity and high mortality rates [43]. Diabetic kidney disease (DKD) affects ~40% of diabetic patients and is the leading cause of ESRD. Treating DKD costs tens of billions of dollars each year and negatively impacts the quality of life for patients and their families. In the United States, minority ethnic groups (e.g., African Americans, Native Americans, and Hispanics) are disproportionately burdened by DKD [44]. Nearly all DKD in African Americans is caused by type 2 diabetes. In addition to environmental influences, type 2 diabetes has a strong genetic component [45–47]. Asymptomatic elevations in urinary albumin excretion and serum creatinine levels, key measures of DKD, are frequently present in diabetic siblings of African American individuals with overt type 2 diabetes [45]. Although DKD is highly prevalent among African American men, the diagnosis and management in this subpopulation has not been well studied. This is due, in part, to the fact that participation in biomedical research among African American men has traditionally been low. Moreover, even when there is equal access to care, diabetic African American men have a higher risk of ESRD than either their Caucasian counterparts or female African American diabetic patients [48]. Despite evidence that genetic factors play a role in the health disparities of DKD, data pertaining to the molecular mechanisms underlying diabetic kidney disease in African Americans—particularly African American men—is lacking. Gaining this information will be important if we are to provide patients with strategies for effective interventions. Proportional representation of all ethnic groups and genders during the analysis of biological samples used for the development of biomarkers of DKD ensures that those with diverse genetic backgrounds are included. This also ensures development of therapeutic targets and diagnostic tools that are efficacious for all patients. Moreover, they may facilitate more targeted interventions in situations where predisposing genetic factors alter the pathology of disease. Previous studies showed that metabolic markers used for diagnosis of the metabolic syndrome, which is associated with a high risk for diabetes, have ethnic differences [49]. Although obesity, insulin resistance, diabetes, and hypertension are more common in African Americans than Caucasians, ethnic differences often lead to underdiagnosis of the metabolic syndrome among African American children and adults. Consequently, many African Americans who are at risk for type 2 diabetes and cardiovascular disease are not diagnosed in a timely manner, which delays the therapeutic interventions that could slow the progression of the disease.

The current study reveals undiagnosed kidney injury in a significant proportion of uninsured and underinsured diabetic African American men in Greensboro, NC (Table 2). Importantly, the data provide insights into a potential role of meprin metalloproteases in the progression of kidney injury in this population. The kidney injury assessment utilized the UACR, which is the gold standard clinical test for DKD, together with a panel of three recently developed protein biomarkers of kidney injury that are not yet clinically available. Microalbuminuria, which occurs due to ultrastructural changes in the glomerular filtration barrier, is the earliest clinical sign of DKD and is positively correlated with age, hypertension, hyperglycemia, smoking, and male gender [50]. However, unlike the UACR, which confirms general kidney injury, the recently developed proteomic biomarkers have the potential to offer insights into the sites of kidney injury. KIM-1 is a type 1 transmembrane glycoprotein expressed in the proximal tubule [9]. Its ectodomain is shed from cells following kidney injury, allowing urinary KIM-1 concentrations to become detectable within 24 hours of tubular necrosis [51]. Urinary KIM-1 levels have been detected after exposure to a variety of nephrotoxic agents, even before the increase of serum creatinine concentrations [51]. For this reason, KIM-1 is considered a sensitive biomarker of acute kidney injury.* In vitro* studies demonstrated that release of soluble KIM-1 is mediated by a metalloprotease [52]. The current study shows that both urinary and plasma KIM-1 correlate with DKD, particularly among diabetic patients with macroalbuminuria. Interestingly, compared to levels of urinary KIM-1, plasma KIM-1 levels appear to correlate with severity of kidney injury more closely (as determined by UACR range). Similarly, urinary NGAL levels correlated with DKD and were significantly elevated among diabetic patients with macroalbuminuria. NGAL is a 25 kDa protein expressed in neutrophils and certain epithelia, including those in the renal tubules. Renal NGAL is released into both urine and plasma and has been shown to be a sensitive biomarker that is predictive of tubular damage in both acute and chronic kidney injury [53, 54]. In fact, in some physiological contexts, NGAL may be a more promising early marker of kidney injury than UACR [55, 56]. In the current study, although we observed significant increases in urinary NGAL among patients already diagnosed with DKD, there were no significant differences between the DKD group and diabetic groups in terms of plasma NGAL levels. Likewise, both urinary and plasma NGAL levels were comparable to controls among patients whose UACR suggested early stages of kidney injury (i.e., microalbuminuria). Interestingly, however, a significant increase in plasma NGAL levels was observed in diabetic patients with macroalbuminuria. Finally, the cysteine-protease inhibitor, cystatin C, has been shown to be a good marker for assessing renal injuries. Urinary cystatin C is considered to be a sensitive marker for the detection of DKD, with levels preceding histopathological changes. Importantly, in previous studies, the levels of cystatin C increased with the progression of renal damage, making it suitable for early detection of kidney injury and accurate assessment of DKD [8, 57]. In the current study, plasma cystatin C levels were significantly elevated in patients with DKD and diabetic patients with macroalbuminuria. Moreover, the elevation of urinary cystatin C levels in patients with DKD was significant (*p* < 0.0001) compared to all the other groups except for the macroalbuminuria subgroup.

This study also suggests that the meprin metalloproteases, meprin A and meprin B, and two kidney meprin targets, nidogen-1 and MCP-1, may play a role in the pathology of DKD in African American men. Previous studies have implicated meprins in the pathophysiology of acute and chronic kidney injury in humans and rodent models of DKD [10, 17]. For instance, single nucleotide polymorphisms (SNPs) in the meprin *β* gene were associated with diabetic kidney injury among the Pima Indians, a US ethnic group with extremely high incidence of type 2 diabetes and diabetic nephropathy [10]. Consistently, meprin expression and activity are decreased in rodents with diabetic kidney injury [16, 17]. In the current study, we detected ~90 kDa protein bands for both meprin A and B in the urine of patients with diabetic kidney injury. Other studies have identified 110 kDa species for meprins, corresponding to the monomeric protein forms [41, 58]. It is possible that the 90 kDa band represents a cleavage product released under pathological conditions. A cleaved meprin fragment of comparable size was reported in kidney proteins from mice subjected to ischemia/reperfusion-induced kidney injury [59]. The fact that meprin protein levels were significantly increased in patients with DKD suggests increased shedding of meprins in diabetic kidney injury. To our knowledge, this is the first study to report increased meprin shedding during human diabetic kidney injury. ADAM10-mediated shedding of meprin A was reported in ischemia/reperfusion and in small intestines [59, 60]. Furthermore, among diabetic patients, the levels of urinary meprins increased with progression from normo- to micro- and macroalbuminuria, suggesting the that meprins may have diagnostic value in detecting diabetic kidney injury. Moreover, increased shedding of meprins from the BBM could have negative pathological consequences in DKD. For instance, previous work from our group showed that meprin-deficient mice with STZ-induced type 1 diabetes had more severe kidney injury when compared to wild-type counterparts [17]. We also documented meprin expression in the glomeruli of diabetic mice, suggesting that they could play a role in both glomerular and tubulointerstitial renal pathology [34].

Knowledge about potential mechanisms by which meprins modulate the pathology of kidney disease is growing. Several studies have identified meprin targets in the kidney, which release proteolytic products into urine. Urinary meprins have previously been proposed as biomarkers of DKD [61]. The current study demonstrates that increased urinary levels for meprins A and B, as well as two meprin targets, nidogen-1 and MCP-1, correlate with progression of DKD. Nidogen-1, an ECM protein, is an important component of the renal basement membrane. Nidogen-1 integrates other membrane components into the ECM and acts as a connecting element between collagen and laminin [62]. Meprins were shown to cleave nidogen-1 and release an approximately 50 kDa fragment in the urine of mice with cisplatin-induced nephrotoxicity [20]. Meanwhile, monocyte chemoattractant protein-1 (MCP-1) is a chemokine that contributes to inflammation by recruiting and trafficking of mononuclear immune cells to sites of inflammation [63].* In vitro* studies have shown that both meprin A and meprin B proteolytically process and cause inactivation of MCP-1 [32]. The current study shows that urinary, but not plasma, levels of MCP-1 increase with kidney injury in diabetic African American men. Previous studies have shown that MCP-1 is a potential marker for predicting progression of DKD [64, 65]. Urinary MCP-1 levels were significantly elevated in patients with DKD and advanced tubulointerstitial lesions [66].

Together, these studies suggest that meprins and several meprin targets could serve as diagnostic tools for the identification of kidney injury in African American men. However, additional large-scale studies are needed to determine the efficacy of these proteins as biomarkers of DKD in this population. Likewise, the utility of meprins and their proteolytic products as diagnostic biomarkers of DKD in other ethnic groups has yet to be explored. Indeed, due to genetic factors contributing to disparities in DKD, new biomarkers are needed for early identification of patients who are prone to the development of DKD. In the age of precision medicine, the development of such biomarkers would facilitate early interventions and thus slow progression to ESRD. Importantly, participants for this study were recruited from a community health and wellness clinic run by Cone Health, the largest healthcare provider in the city of Greensboro, NC. Community clinics play a large role in providing healthcare for uninsured and underinsured patients. The outcomes from this study will enable us to plan community outreach programs that are relevant to African American men in North Carolina.

## Figures and Tables

**(a):** 

	Nondiabetic controls	Diabetics	*p* value
*n*	75	85	
Age (years)	45.3 ± 12.8	49.3 ± 10.1	0.0215
Body mass index (kg/m^2^)	30.5 ± 8.3	32.7 ± 7.9	0.0695
Waist circumstance (cm)	101.0 ± 17.7	112.7 ± 17.3	0.0002
Blood pressure (mmHg)			
Systolic	135.9 ± 20.4	137.4 ± 19.8	0.6236
Diastolic	87.12 ± 12.8	86.2 ± 11.8	0.6224

**(b):** 

	*Nondiabetic controls*	*Diabetics*			*DKD*
		Normoalbuminuria	Microalbuminuria	Macroalbuminuria	
*n*	75	41	27	8	9
Age (years)	45.8 ± 12.8^a^	50.3 ± 11.2^a^	49.7 ± 8.9^a^	46.3 ± 12.2^a^	44.2 ± 8.9^a^
BMI (kg/m^2^)	30.5 ± 7.7^a^	32.9 ± 8.0^a^	32.9 ± 7.5^a^	30.3 ± 6.3^a^	27.5 ± 4.2^a^
WC (cm)	101.0 ± 17.7^b^	114.3 ± 16.8^a^	112.8 ± 17.5^a^	102.6 ± 16.1^ab^	101.3 ± 13.15^ab^
Blood pressure					
Systolic (mmHg)	135.9 ± 20.4^a^	135.2 ± 19.9^a^	135.5 ± 16.4^a^	141.5 ± 32.6^a^	144 ± 21.4^a^
Diastolic (mmHg)	87.12 ± 12.8^a^	85.2 ± 12.0^a^	86.1 ± 12.5^a^	86.1 ± 13.1^a^	89.6 ± 14.5^a^

**Table 2:** Biomarkers of kidney injury in diabetic patients with no known kidney disease, nondiabetic controls, and diabetics with diagnosed kidney disease. DM: diabetes mellitus; DKD: diabetic kidney disease; KIM-1: kidney injury molecule-1; NGAL: neutrophil gelatinase-associated lipocalin; MCP-1: monocyte chemoattractant protein-1; Q3: upper quartile; IQR: interquartile range.

Biomarker	Plasma KIM-1 (pg/ml)	Urine KIM-1 (*μ*g/g Cr)	Plasma cystatin C (*μ*g/ml)	Urine cystatin C (ng/g Cr)	Plasma NGAL (ng/ml)	Urine NGAL (ng/g Cr)
Range of nondiabetic group	(7.78, 63.44)	(0.16, 1.23)	(0.49, 1.21)	(5.29, 82.72)	(5.98, 76.64)	(0.43, 26.57)

Mean ± SD	30.39 ± 17.97	0.47 ± 0.30	0.87 ± 0.20	27.45 ± 20.74	41.75 ± 18.24	5.84 ± 5.71
>mean + 2SD in DM versus in DKD	52% versus 100%	23% versus 20%	17% versus 78%	19% versus 64%	17% versus 40%	24% versus 73%
>mean + 3SD in DM versus in DKD	45% versus 80%	12% versus 20%	11% versus 78%	14% versus 64%	13% versus 40%	21% versus 64%

Q3 ± IQR	46.56 ± 29.62	0.54 ± 0.31	0.97 ± 0.22	27.75 ± 11.95	55.44 ± 27.40	7.55 ± 5.04
>Q3 + 1.5 IQR in DM versus in DKD	40% versus 80%	25% versus 27%	17% versus 78%	43% versus 79%	13% versus 40%	24% versus 73%
>Q3 + 3 IQR in DM versus in DKD	24% v. 70%	10% versus 20%	11% versus 78%	21% versus 64%	6% versus 30%	21% versus 64%
